# Estimation of Survival in Patients with Glioblastoma Using an Online Calculator at a Tertiary-Level Hospital in Mexico

**DOI:** 10.7759/cureus.32693

**Published:** 2022-12-19

**Authors:** María S Aguirre-Madrigal, José G Flores-Vázquez, Gerardo Romero-Luna, Viviana Ramírez-Stubbe, José Javier Morales-Ramírez, Citlali Alfaro-López, Jesús Daniel Rembao-Bojórquez, Sergio Moreno-Jiménez

**Affiliations:** 1 Neurosurgery, Instituto Nacional de Neurología y Neurocirugía Manuel Velasco Suárez, Mexico City, MEX; 2 Escuela de Ciencias de la Salud, Universidad Anáhuac Querétaro, Querétaro, MEX; 3 Neuropathology, Instituto Nacional de Neurología y Neurocirugía Manuel Velasco Suárez, Mexico City, MEX; 4 Neurological Center, ABC Medical Center, México City, MEX

**Keywords:** machine learning, prognosis, prediction, online calculator, overall survival, neurooncology, glioblastoma

## Abstract

Background

The mean survival duration of patients with glioblastoma after diagnosis is 15 months (14-21 months), while progression-free survival is 10 months (+/- one month). Although there are well-defined overall survival statistics for glioblastoma, individual survival prediction remains a challenge. Therefore, there is a need to validate an accessible and cost-effective prognostic tool to provide valuable data for decision-making. This study aims to calculate the mean survival of patients with glioblastoma at a tertiary-level hospital in Mexico using the online glioblastoma survival calculator developed by researchers at Harvard Medical School & Brigham and Women's Hospital and compare it with the actual mean survival.

Methodology

We conducted a retrospective observational study of patients who received a histopathological diagnosis of glioblastoma from the National Institute of Neurology and Neurosurgery "Manuel Velasco Suárez" between 2015 and 2021. We included 50 patients aged 20-83 years, with a tumor size of 15-79 mm, and who had died 30 days after surgery. Patient survival was estimated using the online calculator developed at Harvard Medical School & Brigham and Women's Hospital. The estimated mean survival was then compared with the actual mean survival of the patient. A two-tailed equivalence test for paired samples was performed to conduct this comparison. A value of p < 0.05 was considered significant.

Results

The mean age of the sample was 55.5 years (confidence interval (CI) 95%, 52.61-58.71). The mean tumor size in our sample was 49.12 mm (±14.9mm). We identified a difference between the mean estimated survival and the mean actual survival of -1.37 months (CI 95%; range of -3.7 to +0.9). After setting the inferior (IL) and superior limits (SL) at -3.8 and +3.8 months, respectively, we found that the difference between the mean estimated survival and the actual mean survival is within the equivalence interval (IL: p = 0.0453; SL: p = 0.0002).

Conclusions

The actual survival of patients diagnosed with glioblastoma at the National Institute of Neurology and Neurosurgery was equivalent to the estimated survival calculated by the online prediction calculator developed at Harvard Medical School & Brigham and Women's Hospital. This study validates a practical, cost-effective, and accessible tool for predicting patient survival, contributing to significant support for medical and personal decision-making for glioblastoma management.

## Introduction

Glioblastoma is the most prevalent primary malignant tumor of the central nervous system, accounting for 12-15% of intracranial neoplasms [1,2]. It usually occurs in people over the age of 50, and its incidence in the United States is 12,000 cases per year. Glioblastoma can be categorized into several molecular subgroups based on mutation and DNA methylation in proteins such as TP53, ATRX, and IDH [3]. Additionally, about 5% of these tumors correspond to hereditary syndromes such as Li-Fraumeni syndrome, Turcot syndrome, and neurofibromatosis.

Multimodal treatment with surgery, radiotherapy, and chemotherapy is the best alternative for the management of these patients [4]. However, the median survival of glioblastoma after diagnosis is 15 months (14-21 months), while progression-free survival is 10 months (+/- one month). Although well-defined survival statistics are available for glioblastoma overall, individual survival prediction remains a challenge for clinicians due to the complex nature of the disease and the wide variation of survival in the patient population. Therefore, the need arises to validate a prognostic tool that is both accessible and cost-effective to provide valuable data for clinical, therapeutic, and personal decisions [2,5].

Understanding the factors that influence prognosis and survival in disease is highly relevant for therapeutic decision-making. Nowadays, mathematical and statistical models applied to computational systems have allowed the development of multiple artificial intelligence and machine learning systems for answering complex questions efficiently. Machine learning is responsible for developing algorithms that can learn without having to be programmed explicitly, considering all possible scenarios [6-8].

The application of these systems in medicine has led to the development of several predictive tools for various pathologies. It allows for the integration of multiple clinical, physiological, and sociodemographic variables of a patient to make more accurate probability estimations with greater precision. Knowing the factors that influence the modification of the prognosis and survival of a disease is of great relevance for daily clinical activities, as it facilitates therapeutic decision-making and provides insights into the possible evolution of the disease [8,9].

To address these needs, in 2019, Senders et al. [10] at Harvard Medical School & Brigham and Women’s Hospital created a survival predictor algorithm for glioblastoma patients using the Surveillance Epidemiology and End Results (SEER) database in the United States. Data from 20,821 glioblastoma patients undergoing surgery were included, using which 15 statistical and machine learning algorithms were developed to predict one-year overall survival and plot personalized survival curves based on 13 demographic, socioeconomic, clinical, and radiological factors. Their study showed that the “accelerated failure time” model had superior performance in terms of discrimination, calibration, interpretability, predictive applicability, and computational efficiency compared to any other algorithm, including the widely used Cox proportional hazards regression model. With the data obtained on the variables, this model was used to develop a survival calculator for glioblastoma patients. It is available online (https://cnoc-bwh.shinyapps.io/gbmsurvivalpredictor/) for research aimed at obtaining external validity as a predictive tool [10]. Our study aims to compare the estimated individual survival with the actual survival of patients with glioblastoma at the National Institute of Neurology and Neurology “Manuel Velasco Suárez” using this online glioblastoma survival calculator.

## Materials and methods

We retrospectively studied 50 patients who received a histopathologic diagnosis of glioblastoma from the National Institute of Neurology and Neurosurgery between 2015 and 2021. Patients aged 27-83 years, with a tumor size of 15-79 mm, and who had died 30 days after surgery were included. Subsequently, patient data were obtained through the electronic file and the Carestream Vue Motion v12.1.5.6 image viewer.

The following variables were collected for the patients: sex, age, race, marital status, health insurance, primary tumor location, laterality, extent, size, and type of surgical resection. Tumor size was calculated using the largest diameter obtained on magnetic resonance imaging (MRI) T1-weighted with intravenous contrast. These variables were entered into the online survival prediction calculator (https://cnoc-bwh.shinyapps.io/gbmsurvivalpredictor/) developed by Harvard Medical School & Brigham and Women’s Hospital to obtain the estimated survival for each patient. Similarly, patients’ actual survival was calculated from the time of the histopathologic diagnosis to the date of death, which was consulted in the electronic file or by telephone with the patient’s relatives. Patients who did not have the variables required for the study and did not have a date of death available were excluded from the analysis.

The results obtained were captured and organized in a database in Microsoft Excel 2019 v.2209. The statistical packages Microsoft Minitab 2021 v.21.1 and GraphPad Prism v.9.4.1 were used for statistical analysis. The means of the actual survival and the estimated survival were compared to calculate the equivalence between the two. The measures of central tendency were used to obtain the mean and 95% confidence intervals of both survivals. To compare survival means, we used two-tailed equivalence tests for paired samples with inferior (IL) and superior limits (SL) of -3.8 and + 3.8 months, respectively. There are no specific limits or margin of error described in the literature for glioblastoma prognosis, so we obtained a mean from the studies of other glioblastoma prognostic tools and adjusted it to get the most precise margin possible. We analyzed these studies later in the discussion section. We considered p < 0.05 as significant.

The present study was evaluated and approved by the National Institute of Neurology and Neurosurgery “Manuel Velasco Suárez” research board with the number 13/22 following Section 1 of Article 17 of the Regulations of the General Law of Health on Research for Health. This study is considered risk-free research because a retrospective technique was used to collect information, and no interventions were made at any time. Due to the latter, the participants were not asked to sign an informed consent form. It is stipulated in section IX of Article 22 of the General Law for the Protection of Personal Data in Possession of Obligated Subjects, that is, it is not mandatory to obtain the informed consent of the owner to process their data. Therefore, the information collected for this research was treated with discretion and anonymity. The criteria for the execution of health research projects on human subjects stipulated in the Norma Oficial Mexicana NOM-012-SSA3-2012 were followed at any time.

## Results

Of the 50 patients with a histopathologic diagnosis of glioblastoma included in this study, 48% (24) were female and 52% (26) were male. Only 10% of the patients (5) had medical insurance. The location of the tumor was the temporal lobe at 40% (20), followed by the frontal lobe at 30% (15), the parietal lobe at 14% (7), insular at 8% (4), occipital at 4% (2), and cerebellum and midline (4%, one patient in each). Tumor laterality presented a similar proportion, with 50% of the patients (25) on the right side, 46% (23) on the left side, and only 4% (2) on the midline. The mean age of the sample was 55.5 years (SD 55.66 ± 10.74) (Table 1).

**Table 1:** Clinical and demographic data.

	Patients (n = 50)
Age (mean + SD, years)	55.66 ± 10.74
Sex	
Male	26 (52%)
Female	24 (48%)
Marital status	
Single	23 (46%)
Married	27 (54%)
Medical insurance	
Yes	5 (10%)
No	45 (90%)
Primary location	
Frontal	15 (30%)
Parietal	7 (14%)
Temporal	20 (40%)
Occipital	2 (4%)
Insular	4 (8%)
Midline	1 (2%)
Cerebellum	1 (2%)
Laterality	
Left	23 (46%)
Right	25 (50%)
Midline	2 (4%)
Extent of the tumor	
Ventricles	13 (26%)
Midline	8 (16%)
No extension	29 (58%)
Extent of resection	
Biopsy	1 (2%)
Subtotal resection	39 (78%)
Total resection	10 (20%)
Tumor size (mean + SD, millimeters)	49.12 +/- 14.9

Moreover, 60% of the patients (30) were managed with triple therapy surgery + radiotherapy + chemotherapy, 28% (14) were treated with surgery only, and 12% (6) received surgery + radiotherapy. Of the patients treated with surgery, 78% (39) received partial resection, 20% (10) underwent complete resection, and only 2% (1) had a biopsy (Table 1). The mean tumor size in our sample was 49.12 mm (±14.9 mm).

**Figure 1:**
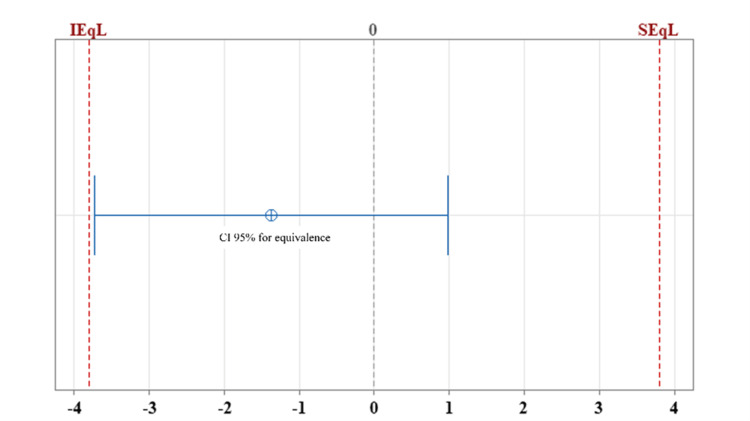
Equivalence test: estimated survival mean – actual survival mean. The 95% confidence interval (CI) for equivalence after calculating the difference between estimated survival mean and actual survival mean ranges from -3.7302 to 0.97865. The CI is within the equivalence interval defined by the inferior and superior equivalence limits (-3.8 and 3.8, respectively); therefore, equivalence can be established. The probability that the estimated survival underestimates the actual survival by < -3.8 months is 4.5%, whereas it overestimates the actual survival by >3.8 months is 0.029%. IEqL: inferior equivalence limit. SEqL: superior equivalence limit

**Figure 2:**
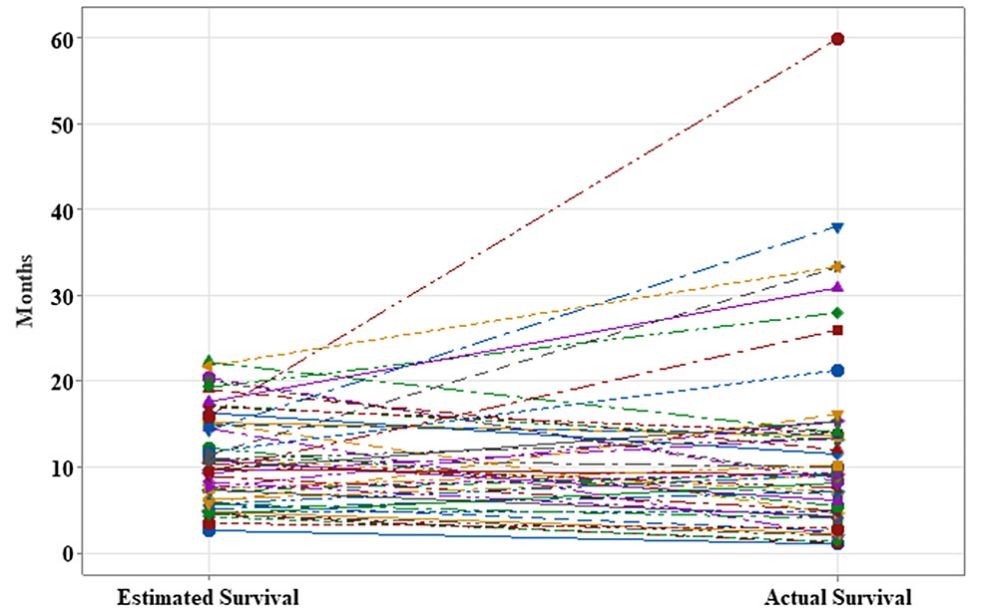
Subject profile plot for equivalence. Every single subject profile of the 50 patients is plotted on this graph. A straight line for each patient represents the estimated and actual survival, showing the difference between them. Most patients had equivalence between survivals, with only eight outliers whose the actual survival was longer than the calculated one.

The mean of the actual survival and estimated survival were 12.18 months (CI 8.97-15.40) and 10.81 (9.27-12.34) months, respectively (Table 2). After applying two-tailed equivalence tests for paired samples, we obtained the difference between the mean estimated survival and the mean actual survival of -1.37 months, with a 95% confidence interval for equivalence ranging from -3.37 months to +0.9 months. After setting the IL and SL at -3.8 and +3.8 months, respectively, we found that the difference between the mean estimated survival and the mean of the actual survival is within the equivalence interval (IL: p = 0.0453; SL: p = 0.0002) (Figure 1, Figure 2). On this basis, the probability of the estimated survival underestimating the actual survival by < -3.8 months is 4.5% and that of the estimated survival overestimating the actual survival by >3.8 months is 0.029%. From the above, equivalence can be affirmed between the survival estimated by the calculator and the actual survival of patients with glioblastoma.

**Table 2:** Descriptive statistics of the actual survival and the estimated survival.

	Actual survival (months)	Estimated survival (months)
Mean	12.18	10.81
95% confidence interval	8.97-15.40	9.27-12.34
Median	9.11	10.05
Variance	128.08	29.07
Standard deviation	11.31	5.39185
Range	1-60	2.6-22.20

## Discussion

Based on the study results, an equivalence between the actual and estimated survivals can be observed, as in Senders et al. [10], who demonstrated a correlation between the estimation of survival through the online calculator and the actual survival of patients with a histopathological diagnosis of glioblastoma. However, Marko et al. [11] showed an underestimated survival of 4.37 months, almost similar to our results; the minor difference is due to the inclusion of additional variables such as clinical presentation and the Karnofsky Performance Status (KPS) in their study.

Other studies, such as Lao et al. [12], have developed tools for predicting survival in patients with a diagnosis of glioblastoma. However, their application involves acquiring and analyzing multiple MRI series within neural networks that are trained for processing. Their tool has a longer processing time and requires equipment with a large storage capacity, which makes its application difficult in institutions that do not have enough time to use and process MRI in a single patient. These researchers performed the validation of their tool using manual measurements in randomly selected patients. When evaluating the results obtained by the algorithm and evaluators, there was a difference of 2.1 months between the actual and estimated survival means, which is close to our mean difference. However, as mentioned above, its use requires multiple technological resources that are not highly available and affordable [12].

Some glioblastoma predictive nomograms have been developed, including several clinical, radiological, and genomic variables. The predictive nomogram developed and validated by the University of Texas MD Anderson Cancer Center includes PTEN, IDH1, and TP53 mutation status, as well as age and KPS. This nomogram calculates the probability of short (< 6 months), median (~ 15 months), or long survival (> 5 years) with a C-index of 0.66. Nevertheless, their mean extent of resection was 100%, making an excellent resection a baseline criterion for its application, which was achievable in only some cases in our context [13]. Gorlia et al. [14] developed multiple nomograms based on variables such as age at diagnosis, WHO performance status (WPS), the extent of resection, mini-mental state examination (MMSE) score, and MGMT methylation status. They achieved a maximum C-index of 0.66, similar to the MD Anderson nomogram. However, this study did not include gender or race and did not conduct an independent validation of the final nomogram. Further, Gittleman et al. [15] built and internally validated a nomogram to predict 6-, 12-, and 24-month survival probabilities, with a C-index of 0.657. The variables in the nomogram included age, gender, KPS, the extent of resection, and MGMT methylation status.

The predictive nomograms mentioned include genomic biomarkers as a significant prognostic factor. Due to their complexity, applicability, and costs, these markers are not routinely clinically employed in many regions, including our center. Specifically, MGMT methylation status is a widely accepted biomarker for sensitivity to temozolomide, directly impacting patient prognosis [15]. However, there is no consensus on optimal measurement methods, and no clear cutoff points have been established. The nomograms presented had baseline inclusion criteria that may not apply to all populations and centers, such as a complete resection and use of current standard treatment. Additionally, there is evidence for a difference in survival by race, but most of the patients included in these studies were white, which may not be applicable in a majorly non-white population such as in our study context.

In contrast, Senders et al. [10] calculator includes race and tumor characteristics, which may allow for a generalizable application of the tool. However, it does not consider the patient’s initial condition with KPS or WPS. Therefore, a direct comparison of results between nomograms, and the calculator is needed to observe the differences between predictions.

In 2011, a predictive algorithm was designed; it includes the extent of tumor resection as the main variable, which is considered the most critical factor for predicting the survival of these patients to the present day. Additionally, the eloquence of the tumor location was included, which may have an implicit operator-dependent bias since, in recent years, certain brain areas have been considered to perform essential functions that were earlier considered non-eloquent areas. This study created a very similar model to that of the calculator used in our study using the “accelerated failure time” algorithm and the statistical analysis through Cox proportional hazards regression model. The difference between actual and estimated survival reported in their study was -5.57 to +5.90 months, which is higher than that reported in our study [16].

Our results show smaller differences between survivals compared to other predictive algorithms previously developed. However, the calculator used in our study includes accessible variables of the patient with a minimum cost that does not imply the use of complex computational systems or genomic tests that, due to time and resource constraints, would not be applicable in many institutions. The findings of this study provide a framework for survival prediction models that offer an individualized and more accurate estimate with accessible variables but without additional cost beyond the minimum necessary for patient medical care. Nonetheless, variables still need to be considered due to their influence on the prognosis and evolution of the patient. Such is the case of the radio-chemotherapy scheme employed or the initial patient condition. Improving the accuracy of the predictions of different models should be performed in future studies since, in a pathology where survival is so short, a difference of months is considered relevant.

## Conclusions

Our study found that the survival of patients with glioblastoma estimated by the online calculator underestimates patients' actual survival by at most -3.8 months. However, it only overestimates actual survival by 0.9 months. Considering this margin of error, we conclude that the estimated survival by the online calculator developed at Harvard Medical School & Brigham and Women's Hospital is equivalent to the actual survival of patients diagnosed with glioblastoma at the National Institute of Neurology and Neurosurgery. This study contributes to the validation of a practical, low-cost, and accessible tool for predicting the survival of patients with glioblastoma, representing important support for medical and personal decision-making for their treatment.

## References

[1] Celis MÁ, Alegría-Loyola MA, González-Aguilar A (2015). Primer consenso mexicano sobre recomendaciones de la atención multidisciplinaria del paciente con glioblastoma multiforme (GBM). Gac Med Mex.

[2] Davis ME (2016). Glioblastoma: overview of disease and treatment. Clin J Oncol Nurs.

[3] Louis DN, Perry A, Wesseling P (2021). The 2021 WHO Classification of Tumors of the Central Nervous System: a summary. Neuro Oncol.

[4] Rodríguez-Camacho A, Flores-Vázquez JG, Moscardini-Martelli J (2022). Glioblastoma treatment: state-of-the-art and future perspectives. Int J Mol Sci.

[5] Tan AC, Ashley DM, López GY, Malinzak M, Friedman HS, Khasraw M (2020). Management of glioblastoma: state of the art and future directions. CA Cancer J Clin.

[6] Sandoval Serrano LJ (2018). Algoritmos de aprendizaje automático para análisis y predicción de datos. REDICCES.

[7] Rajpurkar P, Chen E, Banerjee O, Topol EJ (2022). AI in health and medicine. Nat Med.

[8] Senders JT, Arnaout O, Karhade AV, Dasenbrock HH, Gormley WB, Broekman ML, Smith TR (2018). Natural and artificial intelligence in neurosurgery: a systematic review. Neurosurgery.

[9] Sussman L, Garcia-Robledo JE, Ordóñez-Reyes C (2022). Integration of artificial intelligence and precision oncology in Latin America. Front Med Technol.

[10] Senders JT, Staples P, Mehrtash A (2020). An online calculator for the prediction of survival in glioblastoma patients using classical statistics and machine learning. Neurosurgery.

[11] Marko NF, Weil RJ, Schroeder JL, Lang FF, Suki D, Sawaya RE (2014). Extent of resection of glioblastoma revisited: personalized survival modeling facilitates more accurate survival prediction and supports a maximum-safe-resection approach to surgery. J Clin Oncol.

[12] Lao J, Chen Y, Li ZC, Li Q, Zhang J, Liu J, Zhai G (2017). A deep learning-based radiomics model for prediction of survival in glioblastoma multiforme. Sci Rep.

[13] Ferguson SD, Hodges TR, Majd NK (2021). A validated integrated clinical and molecular glioblastoma long-term survival-predictive nomogram. Neurooncol Adv.

[14] Gorlia T, van den Bent MJ, Hegi ME (2008). Nomograms for predicting survival of patients with newly diagnosed glioblastoma: prognostic factor analysis of EORTC and NCIC trial 26981-22981/CE.3. Lancet Oncol.

[15] Gittleman H, Lim D, Kattan MW (2017). An independently validated nomogram for individualized estimation of survival among patients with newly diagnosed glioblastoma: NRG Oncology RTOG 0525 and 0825. Neuro Oncol.

[16] Sanai N, Polley MY, McDermott MW, Parsa AT, Berger MS (2011). An extent of resection threshold for newly diagnosed glioblastomas. J Neurosurg.

